# CANCER: ARE OFFSPRING OF LONG-LIVED SIBLINGS BOTH ROBUST AND RESILIENT?

**DOI:** 10.1093/geroni/igz038.3314

**Published:** 2019-11-08

**Authors:** Angéline Galvin, Jacob Krabbe Pedersen, Svetlana Ukraintseva, Thomas T Perls, Mary K Wojczynski, Kaare Christensen

**Affiliations:** 1 Epidemiology, Biostatistics, and Biodemography, Department of Public Health, University of Southern Denmark, Odense, Denmark; 2 The Danish Aging Research Center, Department of Public Health, University of Southern Denmark, Odense, Denmark; 3 Center for Population Health and Aging, Duke University, Durham, North Carolina, United States; 4 Boston University School of Medicine, Boston, Massachusetts, United States; 5 Department of Genetics, Washington University in St. Louis, Saint Louis, Missouri, United States; 6 Danish Aging Research Center, University of Southern Denmark, Odense C, Denmark

## Abstract

Background: The mechanisms underlying clustering of longevity in families are unclear. We have previously shown a low cancer incidence in offspring of long-lived siblings, i.e. cancer robustness. Here we test whether such offspring are also more resilient in terms of survival after cancer diagnosis. Methods: Identification of offspring from long-lived families was undertaken in three nationwide, consecutive Danish studies (DOS, GeHA, LLFS). Cancer cases were identified through linkage with the Danish Cancer Registry. Each offspring cancer case was matched with two control cancer cases from the 5% random sample of the Danish population. Matching criteria were birth year, sex, year of diagnosis and cancer site. The main outcome was overall survival. Factors studied were sociodemographic, health-related and cancer-related. Survival analyses were performed using stratified Cox proportional hazards models based on the matching data. Results: Among the 5,377 offspring of the 634 families, 465 offspring of long-lived siblings with first primary cancer were included, along with 930 controls. Offspring of long-lived siblings had a significantly better survival than controls (HR=0.64 95%CI=[0.52-0.78]). The association attenuated only slightly after adjustment of marital status, education, Charlson Comorbidity Index, and number of prescribed drugs (HR=0.66 95%CI=[0.54-0.81]). Conclusion: Our results suggest that in addition to being more robust to cancer risk, offspring of long-lived siblings are also more resilient to cancer after its diagnosis and show better overall survival compared to individuals with cancer from general Danish population. Funding: The LLFS study is funded by the US National Institute on Aging / National Institutes of Health.

